# Army News

**Published:** 1919-01

**Authors:** 


### Army News.

---

Dr. Will C. Duringer, who enlisted in the service several months ago, has been transferred from Camp Greenleaf, Ga., to a reconstruction hospital at Des Moines, Iowa.

Major John O. McReynolds, flight surgeon at Camp Dick, has returned to civilian life, having accepted his discharge from the army.

Several officers from the Base Hospital at Camp Bowie have received honorable discharges from the Army. Among the number discharged are Capt. W. Quigley, Lieuts. T. A. Dickson, Ersner W. West and L. Greenberg.

Major Charles E. Cantrell, U. S. Army Medical Corps, of Greenville, has received his discharge from the army. He was chief surgeon of the U. S. Army General Hospital No. 15 at Corpus Christi.

Lieut. Col. H. P. Carter has been appointed camp surgeon at Camp Travis, being transfered there from the War College. Maj. A. L. Van Meter, who has filled the position of camp surgeon previous to Colonel Carter's appointment, has been appointed to the post of assistant surgeon, Eighteenth Division.

First Lieutenant George L. Carlisle of Dallas, Texas, has sailed from France on his return home and is expected to arrive in New York very soon. A cablegram announcing his departure from France has been received by Mrs. Carlisle. He has been with the Baylor Hospital Unit on duty with the army in France.

Dr. G. D. Parker has returned from Camp McArthur, where he served in the medical reserve corps in the central officers' training school hospital. Dr. Parker is remaining in the medical reserve corps subject to call in case of emergency, but for the present will resume his practice in Houston.

Demobilization of the entire personnel of Camp Greenleaf, the medical corps training camp was ordered December 17th by Colonel W. N. Bisham, commanding, after the camp had been inspected by Surgeon Ireland. There are about 8,000 men at the camp. It was announced that most of the enlisted men would be mustered out at once, officers of the regular establishment would be transferred to various base and general hospitals and reserve officers except under 38 years old who wish to remain in the army would be discharged from service.

Dr. J. E. Gilcreest, of Gainesville, Texas, has received word that his son, Dr. Edgar Gilcreest, who was practicing in Dallas at the time of his enlistment in the Army Medical Corps, has been promoted to Major and is likely to remain abroad another year. Major Gilcreest has seen several years of war service, having served as surgeon during the Balkan war.

A letter of appreciation for the services rendered the country has been received by the Medical Advisory Board of Dallas from Major W. B. Russ, medical aid to Governor W. P. Hobby. The communication, which was addressed to Dr. E. H. Cary, stated that the State of Texas tied for fourth place with Delaware in the smallest per cent of men returned from cantonments and training camps through faulty examinations made by local boards and medical advisory boards. In addition, Major Russ said that the officials at Washington in considering the work of each state, had remarked that Texas had done the best work of all, as it had a large amount of rural districts to cover. Figures quoted on percentage of men returned because of desultory examination upon registration were: Pennsylvania, 8.45; Ohio, 6.29; New York, 7.59; Texas, 4.59.

"Over 4,500 men passed through the local board of Dallas. The Advisory Board is looked upon as above the average of State boards and has done a greater amount of work, as it had to deal with most thickly populated districts of the State," Dr. Gary said.

Men on the board gave three nights each week entirely to Government work and were faithful to every engagement.

